# Families Flourish: Triangulating Housing, Neighborhood, and Life Coaching for Health

**DOI:** 10.3390/ijerph23060724

**Published:** 2026-05-29

**Authors:** Jason Reece, Jee Young Lee, Rachel Kleit

**Affiliations:** City & Regional Planning, Knowlton School, Ohio State University, Columbus, OH 43210, USA; lee.8000@osu.edu (J.Y.L.); kleit.1@osu.edu (R.K.)

**Keywords:** housing mobility, fair housing, mental health, neighborhood health, healthy housing, neighborhood safety, indoor health hazards

## Abstract

**Highlights:**

**Public health relevance—How does this work relate to a public health issue?**

- Housing quality and neighborhood conditions exert a profound influence on child health and other life outcomes. Within the U.S. housing market, low-income children are disproportionately likely to reside in distressed housing and neighborhood environments, and the risk is even greater for children from marginalized racial and ethnic populations.
- Although the literature on housing quality and child health is extensive, relatively few studies explicate the mechanisms by which fair housing strategies—such as housing mobility—directly affect child and family health.

**Public health significance—Why is this work of significance to public health?**

- Families Flourish participants reported a wide range of positive health outcomes, driven by safer housing, reduced stress, supportive coaching, improved financial stability, and access to better school environments. Together, these factors enabled families to promote healthier lifestyles, better manage chronic conditions, and enhance emotional well-being across generations.
- Data suggests several descriptive pathways associated with perceived housing and neighborhood improvements into improved family health, unfolding over time. Immediate improvements to indoor air quality and neighborhood safety improved respiratory health outcomes and facilitated more outdoor activity and play. Reduced family and child stress improved mental health outcomes for children and improved parenting style and parental engagement. Participation in a well-resourced school system provided more direct support from educators and supportive services for children.

**Public health implications—What are the key implications or messages for practitioners, policy makers and/or researchers in public health?**

- In planning for family health, fair housing mobility programs are one specific tool that can improve health outcomes. The experience of Families Flourish indicates that improvement to children’s health and educational outcomes occurs sequentially over time. Our research suggests that a three-year subsidy may be sufficient to support housing long enough to produce stability and economic improvement for families in this study.
- Fair housing mobility policies should complement other strategies that improve the overall supply of affordable healthy housing and planning efforts to improve environmental conditions and resources in disadvantaged neighborhoods. Fair housing mobility programs should also be aligned with efforts to reduce exclusionary housing barriers (particularly regulatory barriers such as exclusionary zoning) in safe, healthy, and highly resourced neighborhoods. More importantly, planning for family health requires prioritizing the agency and preferences of families marginalized by the housing market.

**Abstract:**

Previous research demonstrates that housing security and quality influence physical and mental health. Despite a rich literature on housing and health, less is known about the processes through which housing mobility programs directly affect family health. We use a single-case design to examine how the health of families with children is impacted by Families Flourish, a mobility program that combines three years of rental assistance with life coaching and placement in safe, well-resourced neighborhoods. Drawing on developmental and formative evaluation data, including longitudinally collected surveys, interviews, and administrative records, we trace families’ experiences over time. Our analysis identifies distinct pathways through which mobility improves mental and physical health—via improved indoor air quality, reduced environmental and parental stress, and enhanced access to resources. Initial health gains are subsequently leveraged to improve educational and economic outcomes. We observe a temporal sequence in outcomes, with early physical health gains and later mental health improvements as stability and safety increase. We conclude by situating these identified pathways within existing scholarship and discussing implications for planning and fair housing practice.

## 1. Introduction

Housing quality and neighborhood conditions exert a profound influence on child health and other life outcomes [1,2,3,4,5]. Within the U.S. housing market, low-income children are disproportionately likely to reside in distressed housing and neighborhood environments, and the risk is even greater for children from marginalized racial and ethnic populations [6,7]. Although the literature on housing quality and child health is extensive [1], relatively few studies explicate the mechanisms by which fair housing strategies—such as housing mobility—directly affect child and family health.

Housing mobility programs expand opportunities for lower-income families to relocate from distressed areas to safer, higher-resource neighborhoods by leveraging various forms of rental assistance. These programs aim to remediate fair housing barriers that constrain access to opportunities for low-income households and communities of color.

Fair housing interventions arose in response to both private- and public-sector discrimination that historically shaped racially segregated housing markets in the United States [8]. Despite the Fair Housing Act of 1968 and subsequent reforms, exclusionary housing and land-use policies continue to restrict the supply of affordable—even moderate-income—housing in many metropolitan suburbs and exurbs, while discrimination persists for low-income households, renters with children, and racial and ethnic minorities [9,10]. Although mobility programs remain limited in scale, prior research has documented a range of positive outcomes, with especially strong long-term economic benefits for children who move to lower-poverty neighborhoods [11].

This case study analyzes the mechanisms shaping physical, mental, and emotional health improvements among families participating in Families Flourish, a housing mobility program in Columbus, Ohio. Families Flourish provides access to safe housing in highly resourced neighborhoods and pairs housing assistance with intensive life coaching. Participants volunteer for three years of coaching and programming while receiving privately funded rental assistance for market-rate units in high-opportunity neighborhoods across the metropolitan region.

Drawing on longitudinally collected data from 52 of the 55 enrolled families, we identify key pathways through which mobility may be associated with health improvement. We find temporal sequencing in outcomes: early physical health gains, particularly among children, emerge soon after relocation; as families establish stability, enhanced safety and security facilitate improvements in mental health. We conclude by discussing how these descriptive pathways intersect with the existing literature and the implications for planning to support child and family health.

## 2. Literature Review: Housing Mobility and Health

A substantial body of case studies and longitudinal research examines outcomes for adults and youth in housing mobility programs, including the U.S. Department of Housing and Urban Development’s multi-city Moving to Opportunity (MTO) demonstration [12]. MTO randomly assigned families to one of three groups: an experimental group receiving assistance to lease in low-poverty neighborhoods (poverty < 10%) plus counseling; a second group receiving portable vouchers without locational restrictions or counseling; and a control group remaining in traditional assisted housing. Additional evidence derives from smaller mobility programs that emerged from fair housing litigation in several metropolitan areas [13].

### 2.1. Housing Unit and Neighborhood Effects on Health

Syntheses of mobility programs—from small litigation-driven efforts (e.g., Yonkers, NY, USA; Chicago, IL, USA) to MTO—document improvements in youth health, including lower rates of substance use, depression, behavioral problems, malnourishment, and respiratory disease. Mobility likely operates through concurrent improvements at the unit and neighborhood scales [14]: better housing quality reduces exposure to pests, mold, moisture, and other indoor environmental hazards, while safer neighborhood conditions reduce environmental stress, particularly through enhanced perceptions of safety [15,16].

### 2.2. Reduced Exposure to Violence and Stress

Exposure to violence is a salient neighborhood mechanism affecting both physical and mental health in children. The benefits of relocation out of Boston’s public housing projects produced a decrease in behavioral problems for youth (particularly for boys) and improved mental health for both children and parents [17]. Evidence from MTO indicates that relocation to safer environments produced rapid benefits, including reductions in behavioral problems and improvements in mental health for both parents and children. A 2009 review of the Boston and New York MTO experiment outcomes also identified improved safety and improved mental health as a primary outcome of housing mobility [18]. Parents leaving high-crime public housing described living “life on watch,” marked by hypervigilance and social isolation, which eased after moving to safer communities [17].

### 2.3. Reduced Stress and Parenting

Family stress associated with high-poverty, unsafe neighborhoods is linked to more frequent interpersonal conflict and harsher parenting strategies, which may be protective responses to resource deprivation and violence exposure. Reductions in environmental stress after relocation can therefore translate into less punitive parenting and improved family dynamics [19].

### 2.4. Social Mixing Versus Relative Deprivation

Policies promoting mixed-income neighborhoods often presume role-model or peer effects. However, conflicting findings suggest that “relative deprivation”—heightened psychosocial stress due to visible income differences—may offset benefits for some youth who move to more affluent areas, with documented associations to depression, social phobia, aggression, and parent–child conflict [20,21]. Racial bias and differential surveillance may compound these effects, particularly for older boys. Recent longitudinal research generated from the HOPE VI mixed-income housing redevelopment program counters these earlier studies, demonstrating significant longitudinal benefits for children living in the mixed-income developments compared to their peers in predominantly low-income communities [22].

### 2.5. Gender and Age Differences in Outcomes

Mental health outcomes in mobility programs often differ by gender and age. Girls tend to report improved safety, mental health, and social integration following moves to lower-poverty neighborhoods, whereas older boys may experience fewer benefits or even negative outcomes, potentially related to reduced access to same-gender role models and heightened perceptions of police surveillance in new neighborhoods [23].

## 3. Case Background and Methods

Building on prior evidence of benefits from mobility, we adopted a single-case study approach to illuminate the processes by which families experience change. The design aligns with Yin’s descriptive and explanatory case categories and with best practices in case analysis for planning, emphasizing action orientation, a specific population (lower-income, predominantly female-headed families with children), and a phenomenon of broad scholarly relevance (housing mobility) [24].

Data are derived from an ongoing formative evaluation of Families Flourish. We examined the following: (1) how family mental and physical health change over a three-year period; (2) the mechanisms—housing conditions, neighborhood environment, coaching, and social support—that influence outcomes; (3) shifts in children’s educational and behavioral outcomes; and (4) how families adjust to new neighborhoods and engage local resources.

To strengthen trustworthiness, we employed methods of triangulation, prolonged engagement, persistent observation, and member checking. Data sources included longitudinally collected surveys, semi-structured participant interviews, administrative records, and participant observation. Survey and interview questions were developed based on existing instruments used in prior housing mobility research. The Ohio State University Institutional Review Board determined the research exempt. Participants received gift-card incentives for surveys and interviews. Study instruments are available at https://www.familiesflourish.org/reports (accessed on 11 April 2026).

### 3.1. Longitudinal Survey Data

Annual surveys assessed baseline and follow-up conditions related to financial well-being, employment, housing quality, and physical and mental health. Fifty-two of fifty-five enrolled families completed at least one annual survey. Participants entered in cohorts of 15–17 families every six months, necessitating staggered survey timing to ensure similar exposure to the program at each wave; surveys were completed at approximately one year (Year 1) and two years (Year 2) post-move. Data collection occurred approximately every six months for each group between October 2023 and March 2025. Twenty-six of 27 families in Groups 1 and 2 completed two annual surveys owing to the longer program duration. An additional 26 of 28 families in Groups 3 and 4 completed one annual survey. Nine families exited early; their responses were excluded from the analysis. In this study, we analyzed Year 1 data from Groups 3 and 4 and Year 2 data from Groups 1 and 2.

### 3.2. Participant Interviews and Administrative Data

Semi-structured interviews with 25 program families over two years (10 of 14 participants in Group 1 at Year 1 and 15 of 26 participants in Groups 1 and 2 at Year 2) explored economic conditions, housing, physical and mental health, and program experiences. Interviews prioritized families with the longest program exposure and were conducted by members of the research team. We reviewed administrative program data, including baseline intake surveys and coach-collected data for the previous three years, including children’s literacy and reading outcomes.

### 3.3. Program Overview

Families Flourish serves low-income households with children under age 13 who are experiencing housing instability or homelessness. Over a three-year period, the program provides rental assistance in high-resource neighborhoods, intensive life coaching, and monthly workshops organized around four pillars: housing stability, financial literacy, education and career, and health and wellness. The program aims to improve children’s academic performance and families’ financial, physical, and mental well-being.

At full capacity, Families Flourish enrolls approximately 100 families, with cohorts of 15–17 families entering and exiting every six months. This analysis covers the first four cohorts (55 families; n = 52 respondents). At the time of data collection, two cohorts (Groups 1 and 2) were nearing completion of their second year and two (Groups 3 and 4) had completed their first year.

Rental assistance supports healthy units in high-opportunity or high-resource neighborhoods. Participating landlords voluntarily relax screening criteria to accommodate families with lower credit scores or incomes. Units must be located in areas rated as having high or moderate opportunity based on the Ohio Housing Finance Agency (OHFA) Opportunity Map, which evaluates census-tract conditions across education, job access, neighborhood well-being, and health.

### 3.4. Participant Profile

Applicants complete an extensive process involving eligibility screening, staff interviews, and a background check. Staff prioritize families with substantial needs who also demonstrate strong motivation to engage in coaching and workshops. The pool of applicant families typically exceeds 1000 households.

The study population comprised 53 single-female-headed households and two couple households (55 households; 57 adults). Survey data were collected from female-headed households only. Children enrolled in the program across Groups 1 through 4 were included in the study population, with child health outcomes assessed using parent-reported data. Eligibility required meeting income thresholds, passing a criminal background check, and not currently using a Housing Choice Voucher. Most families previously resided in low-opportunity neighborhoods, experienced housing instability, or had periods of homelessness. Most participants were employed full-time; average household income was approximately $35,060. Fifty-seven percent of participants had credit scores below 580, complicating access to quality rentals. Approximately 71% reported some post-secondary education.

Nearly half (25/55) were homeless or without their own space (doubled up or renting a room) at entry. Participants were racially diverse: 46 identified as Black, five as White, one as another race, and three as multiracial. Ages ranged from 19 to 43 at entry (mean ≈ 29). Participants had one to three children (average 1.5) aged 13 or younger.

**Figure 1:**
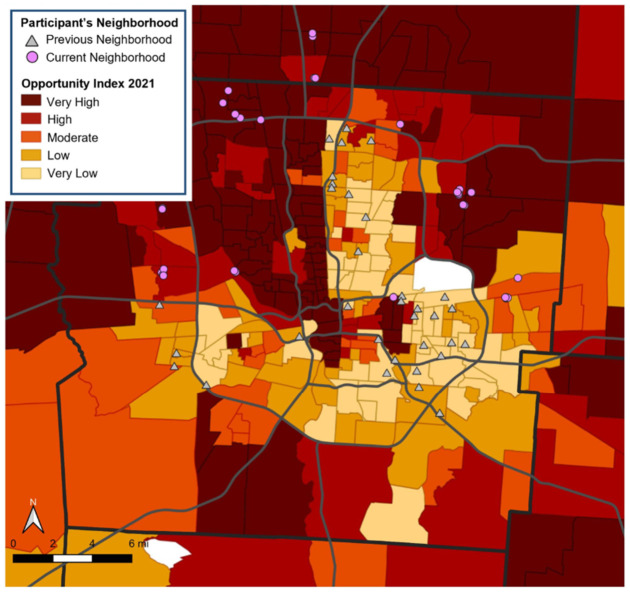
Pre- and post-residential locations for families who were not previously homeless or “doubled up” overlaid with the OHFA neighborhood opportunity index. Areas in white on the map are non residential areas that are not mapped by the opportunity index.

**Figure 2:**
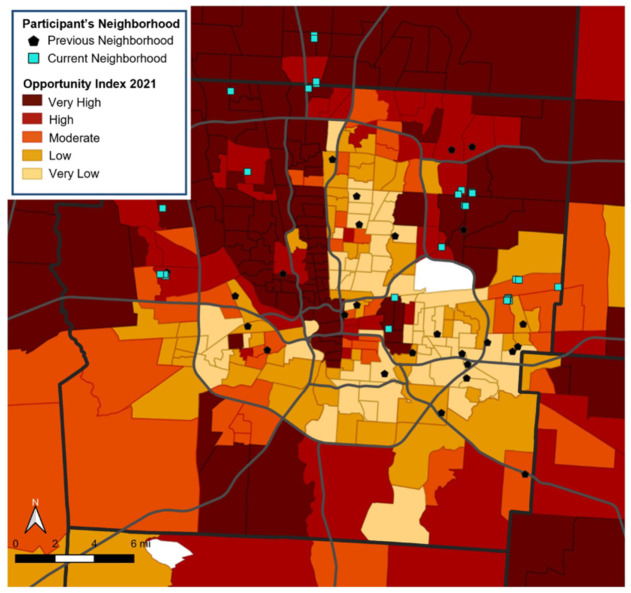
Pre- and post-residential locations for families who were previously homeless or “doubled up” overlaid with the OHFA neighborhood opportunity index. Areas in white on the map are non residential areas that are not mapped by the opportunity index.

Families moved from low-opportunity neighborhoods (per the OHFA index) to high-opportunity areas across the region. Figure 1 and Figure 2 map pre- and post-move locations for (1) families who were not previously homeless or doubled up and (2) families who were previously homeless or doubled up.

### 3.5. Survey Measures and Analytic Approach

Survey and interview questions were developed based on existing instruments used in prior housing mobility research. Survey measures assessed perceived changes in physical health, mental health, and experiences of stress since relocation. Adult participants were asked to report how each outcome had changed since relocating, using categorical response options indicating positive change, no change, or negative change.

Similar items were used to assess perceived changes in children’s health and well-being, based on parent-reported responses. The survey questions and corresponding response categories for each outcome are presented in the Results section.

Analyses were descriptive and focused on summarizing perceived changes in health outcomes. Responses were grouped into positive, neutral, or negative change categories and summarized using frequencies and percentages. Percentages are calculated using available (non-missing) responses; denominators therefore vary slightly across components.

Interviews were conducted online for approximately 30 min. Interview data were analyzed thematically to contextualize survey findings and to identify factors influencing reported changes in health and well-being. Qualitative quotes are presented illustratively to provide contextual insight into participants’ experiences rather than to support comparisons across groups. Accordingly, group identifiers are not reported for individual quotations. Temporal sequencing was examined descriptively by comparing patterns in outcomes over time. Analyses were exploratory and not inferential.

In supplementary analyses, we conducted descriptive within-participant comparisons for participants with data at both Year 1 and Year 2. These analyses were used to examine temporal patterns and to assess whether cohort-based findings were consistent when comparing outcomes within the same individuals over time.

## 4. Results

Survey data were utilized to assess changes in physical and mental health and experiences with stress. The study population comprised 53 single-female-headed households and two couple households, with data available from 52 households (26 in Groups 1 and 2 and 26 in Groups 3 and 4). Parents also responded on behalf of their children to document changes in child health status for each child in the program. Among 81 children enrolled in Groups 1 through 4, parent-reported data were available for 79 children (38 in Groups 1 and 2 and 41 in Groups 3 and 4). Interview data was utilized to better understand factors influencing changes in health status for adults and children.

Table 1 presents applicant characteristics for Groups 1–2 and Groups 3–4 at program entry. Median age, household size, and estimated gross income were similar across groups, while median credit scores and the proportion of participants with some post-secondary education varied across groups.

### 4.1. Change in Health Status—Adults

**Figure 3:**
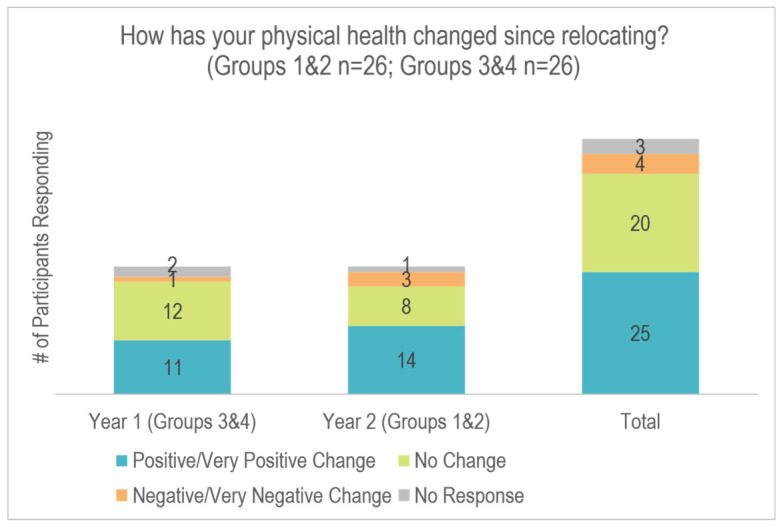
Changes in physical health status reported by adults. Note: “#” refers to “number” of participants.

**Figure 4:**
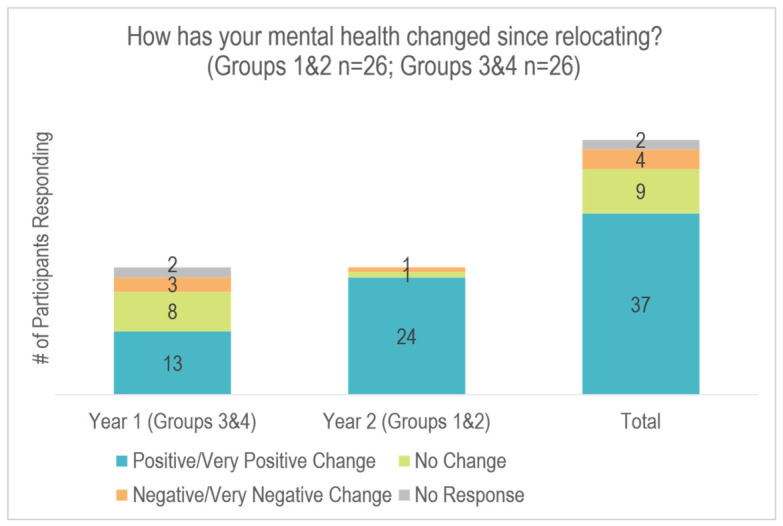
Changes in mental health status for adults. Note: “#” refers to “number” of participants.

**Figure 5:**
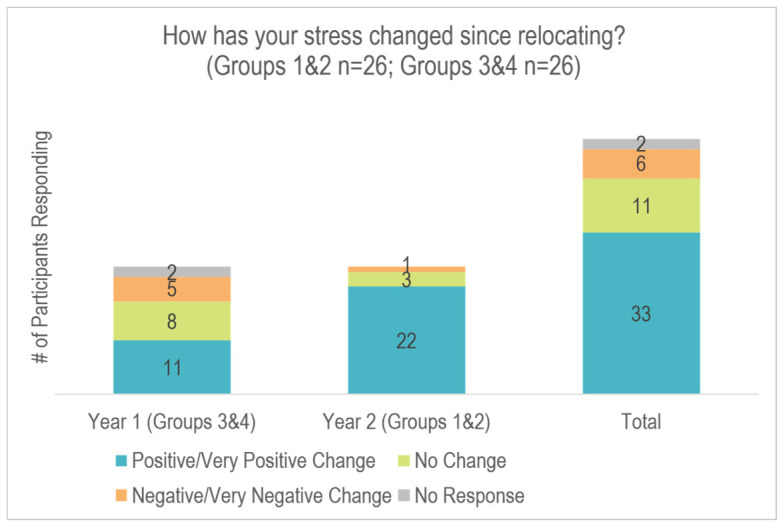
Changes in level of stress reported for adults. Note: “#” refers to “number” of participants.

Just over half of adults indicated a positive change in their health status (25 out of 49 participants with available data; 51%) since joining the program and relocating (Figure 3). Far more adult participants indicated a positive change to their mental health status (37 out of 50 participants with available data; 74%) (Figure 4). Similar findings were reported for changes in levels of stress for adult participants, with 33 out of 50 participants with available data (66%) reporting positive changes to their level of stress (Figure 5). Few adult participants noted a negative change in their physical health (4 out of 49 participants with available data), mental health (4 out of 50 participants with available data), or level of stress (6 out of 50 participants with available data). Time in the program had an impact on the perceived changes in health status and stress. Participants who had completed their second year of the program (Groups 1 and 2) reported higher rates of health and stress improvement than their peers who had just completed one year of the program (Groups 3 and 4).

### 4.2. Change in Health Status—Children

**Figure 6:**
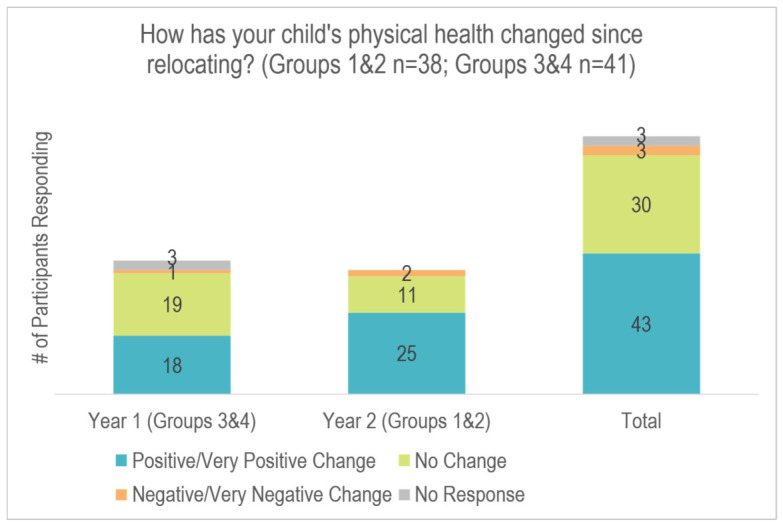
Changes in physical health for children. Note: “#” refers to “number” of participants.

**Figure 7:**
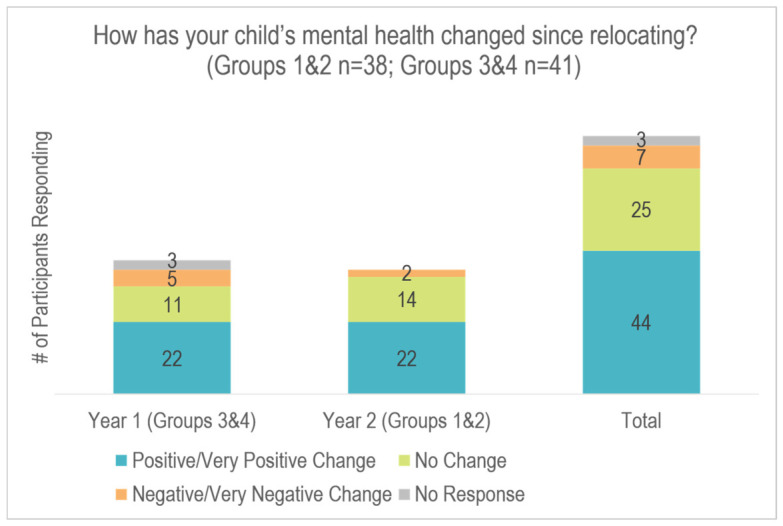
Changes in mental health for children. Note: “#” refers to “number” of participants.

**Figure 8:**
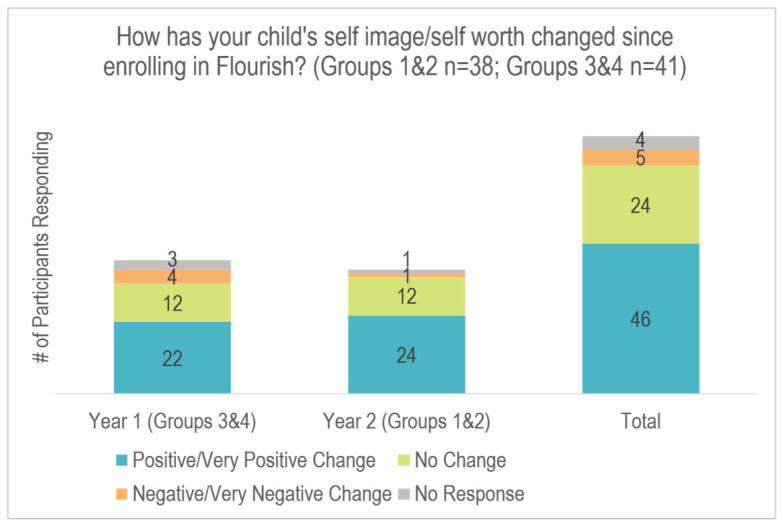
Changes in child’s self-image/self-worth for enrolled children. Note: “#” refers to “number” of participants.

**Figure 9:**
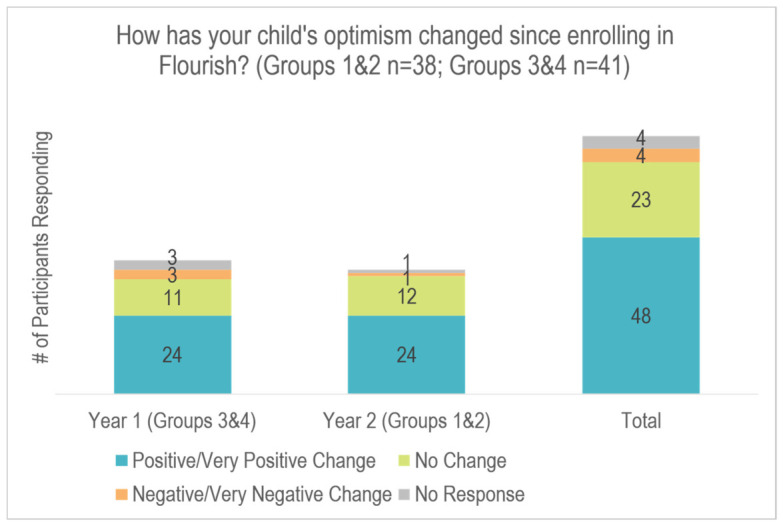
Changes in child’s perceived optimism for enrolled children. Note: “#” refers to “number” of participants.

**Figure 10:**
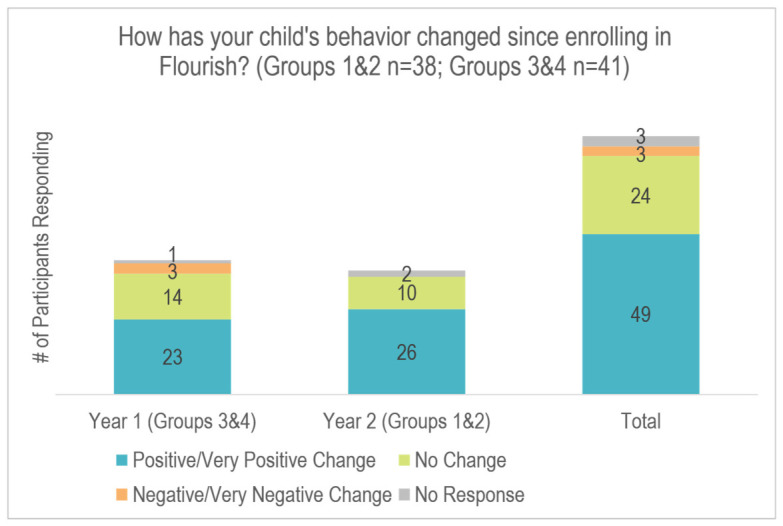
Changes in child behavior for enrolled children. Note: “#” refers to “number” of participants.

Parents reported improved physical health status for more than half of the children enrolled in the program (43 out of 76 children with available data; 57%) (Figure 6). Similar outcomes were reported for changes to children’s mental health (Figure 7), with more than half reporting improvements in mental health (44 out of 76 children with available data; 58%). Similar to adult physical health changes, time in the program was more likely to be associated with a higher proportion of children reported as having physical health improvements. Parents also reported that most children experienced positive changes to their emotional health (Figure 8 and Figure 9) and behavior (Figure 10) since joining the program and relocating. Parents indicated that 46 out of 75 children with available data (61%) experienced positive changes to their children’s self-image/self-worth and noted improvement in their children’s optimism for 48 out of 75 children with available data (64%) in the program. Parents reported positive changes in behavior for 49 out of 76 children with available data (64%).

To assess whether observed patterns reflected changes over time among the same participants, we conducted a supplementary within-participant comparison of Year 1 and Year 2 outcomes (Supplementary Materials). Findings from this analysis were broadly consistent with the primary cohort-based results and provide additional context for observed temporal patterns.

### 4.3. Factors Impacting Change in Health Status: Insights from Participant Interviews

Interview data indicate that participation in Families Flourish contributed to meaningful improvements in both mental and physical health for many families. Parents frequently described reductions in stress, improvements in mood, increased health-promoting routines, and greater engagement with healthcare. Children likewise experienced improvements—especially related to emotional well-being, asthma and respiratory symptoms, and school-based behavioral support. These improvements were often tied to safer neighborhoods, more stable housing, increased resources, and the supportive relationships they formed with coaches and program staff. While a small number of participants reported no changes or ongoing chronic conditions, the dominant pattern reflects increased stability and healthier daily environments.

As illustrated in the following statements, safety was an underlying theme dominating the perspective of parents.

“…the safety and security that I have. I really do appreciate (it), you know, that sense of, security where I know my son and I will be okay.”

“Like, kids can literally be kids out here and you don’t have to worry, “Oh, are they being mean to her?” or “Are they sharing a swing?” Like out here? They literally play. And in the summertime, you can open the windows and you just hear laughing, and, you know, like balls bouncing. You didn’t hear that at my old neighborhood.”

“Oh, my gosh. My old neighborhood, I wouldn’t even let my daughter go outside.”

“So, my new neighborhood is only, like, 7 min from my old neighborhood. And it’s a world apart. Like, it’s so night and day. So, of course, my neighborhood that I live in now is a safer neighborhood. I do live in a secure building, so I just feel safer there. I live near the police station. And because it’s a small community, the police officers, like, you know, my kid goes to school with their kid and we might see them in the store. So, it’s like, he’s our friend, but he’s a protector and a provider and things like that in our neighborhood too. So, I actually see—I didn’t think about, but I actually see policing in a different way living in this neighborhood. So, I definitely feel safer. I feel happier.”

### 4.4. Parental Mental Health

A strong theme across interviews was reduced stress and anxiety, particularly as parents moved into safer environments and gained more stability. Participants explicitly described mental health improvements. Many emphasized a newfound sense of peace, calm, and security, which directly influenced their emotional well-being:

“Yeah, my mental health improved… I’m at peace, you know, knowing I’m safe with 3 kids here by myself.”

“I’m less stressed in this neighborhood… Where I used to live you would hear gunshots. Out here, I haven’t heard any gunshots or anything like that.”

Participants described how improvements in other areas of life—finances, work, access to resources—translated into improved mental health:

“My mental health has gotten a lot better… the rest of my life is starting to fall into place, my anxiety has gotten better, and my depression.”

Others pointed to the program’s coaching support as a mechanism that helped them better manage stress and navigate challenges:

“I’ve learned how to navigate through issues without letting it beat you down.”

Several parents emphasized that reduced personal stress had a direct positive impact on their parenting—and consequently, on their children’s well-being.

“With the program I was able to be the mom that I always wanted… I have less to worry about. I have a safe neighborhood.”

“I still love my neighborhood… My kids are able to go outside and play. They’re able to make friends, and I don’t have to worry.”

“I’m looking forward to just continuing to create moments for my children and myself.”

This improved emotional bandwidth allowed some parents to strengthen relationships with their children, spend more quality time together, or support educational routines such as shared reading.

### 4.5. Child Mental Health

Several interview participants described improvements in their child’s mental or emotional well-being over the past year. These improvements were typically linked to safer neighborhoods, higher-quality schools, and consistent social connections. For example, one parent noted that her child’s overall well-being improved simply by feeling comfortable and socially engaged:

“It’s definitely gotten better… He actually enjoys living in (the suburb of) Gahanna. All his friends—he’s one of those social butterfly kids.”

School-based mental health and behavioral support also contributed to children’s emotional development:

“They just try to find stressors… He has a counselor at his school that talks to him about calming down and how to treat your friends.”

Parents reported improvements related to health support in school, for example, noting the benefits of ADHD support in the new school:

“I feel like the new school deals a lot with ADHD… They have more help.”

These narratives suggest a dual pathway to children’s improved mental health: environmental stability and institutional support.

### 4.6. Parent Physical Health

In interviews, participants (parents) also described progress in their own physical well-being. For some, enhanced motivation and accountability, often supported by their coach, played a key role:

“My goal is to get healthier this year and do more exercising… My mentor said, ‘Let’s hit the gym… or work out at home.’”

Weight loss and increased physical activity emerged as meaningful outcomes:

“I lost 16 pounds… overall at this point, 20 pounds.”

Others linked improvements to increased access to preventive healthcare and consistent medical visits:

“Since relocating, I’m doing better about going to the doctor and addressing issues… I’m doing way better about seeing my providers.”

Participants also noted a significant change in managing chronic conditions, for example,

“My blood pressure… has gone down really tremendously from what it was.”

A small number of interview participants reported no change to their physical health or were managing chronic issues that predated program involvement.

### 4.7. Child Physical Health

In interviews, participants described noticeable improvements in their children’s physical health, especially related to asthma, respiratory issues, or general illness frequency. Moving to newer or cleaner housing conditions was described as a major factor:

“We haven’t had any more asthma issues… At his old apartment, it triggered his asthma a lot more than it does now.”

“She gets sick a lot less… Other places had mold issues and she would always have a cough. Here she only gets sick once or twice a year.”

Families also benefited from better coordination between schools and health needs:

“They have a program where children send his asthma inhaler to the school, so the nurse is able to give it to him.”

Participants who reported no child health changes still noted that their children were “doing well” and staying healthy.

### 4.8. Participant Goal Achievement and Coaching Experiences

Survey findings indicate that most participants (45 out of 52; 87%) reported that the program helped them achieve their goals. Similarly, interview data suggested that participants generally had positive experiences with their coaches and the coaching process. Coaching activities emphasized setting personal goals and developing actionable plans to achieve them. These goals spanned multiple domains, including career development, education, and financial stability. The following excerpts from survey responses illustrate participants’ reported progress toward their goals.

“I’ve been able to live on my own and become more independent.”

“I’ve reached some financial goals and personal goals of my own.”

“I got a better job with higher pay and managing money better.”

“Higher wages.”

## 5. Discussion

A longitudinal perspective on Families Flourish participants reveals a sequence of change. After relocating to healthy, affordable homes in safer, higher-resource neighborhoods, parents reported reduced stress stemming from fewer concerns about safety and greater housing stability. This reduction in stress supported improvements in mental health and enabled deeper engagement with coaching. Together, reduced stress and coaching were associated with substantial economic gains, which appeared to accrue gradually over time, suggesting that a three-year period may be sufficient to support progress toward economic mobility in the context of this study.

### 5.1. Pathways to Health Improvement and Downstream Impacts

An analysis of longitudinally collected qualitative data suggests several descriptive pathways associated with perceived improvements in family health, unfolding over time. First, improved housing quality, especially healthier indoor environments, was associated with reductions in asthma and other respiratory problems for children within months of moving. Second, safer neighborhoods and access to safe outdoor spaces supported children’s physical activity and, along with enhanced perceptions of safety, benefited mental health for both parents and children. Third, by the end of the first year, reduced parental stress enabled shifts toward more effective, less punitive parenting. Fourth, well-resourced schools provided stronger educational and behavioral support tailored to individual needs. Finally, as these conditions stabilized, children’s educational performance improved, with more families later reporting gains in grades and identification for advanced supports.

### 5.2. The Influence of Environment: What Neighborhood Factors Matter Most?

Research indicates a variety of neighborhood factors have an impact on family well-being, quality of life, and health and wellness. Fewer studies directly capture the perspective of these environmental factors for families who are economically marginalized in the housing market and living in distressed environments. The data from Families Flourish participants suggest that certain environmental conditions are closely linked to perceived health and well-being.

In the context of Families Flourish participants, multiple dimensions of neighborhood safety were critical to the benefits of relocation to better neighborhood environments. As articulated by participants, unsafe environments stifled child development, isolated children, and did not allow them to flourish or be active. Safety fears were a primary stressor facing parents, resulting in chronic stress and anxiety. Improving neighborhood conditions can be complex and challenging. Policymakers may focus on a variety of neighborhood improvements (food access, economic development, green space, etc.) and determining how to intervene with limited resources can limit effectiveness. As we approach efforts to improve neighborhood environments, policymakers should integrate the perspectives of families most impacted by challenging neighborhood environments.

### 5.3. Coaching Leverages Environmental Gains

Participants also acknowledged the importance of coaching components of the program. Coaching included programs on parenting, school engagement, and practicing wellness; these skills were instrumental in reducing stress and improving their parenting approach. Parents indicated that they were less likely to lose their patience and could engage more with their children. They were more likely to engage in wellness practices and seek preventative healthcare. Coaching leverages the benefits of reduced stress and improved environmental conditions to support personal growth and development.

### 5.4. Interpreting Outcomes in Relation to MTO and Other Mobility Programs

MTO (Moving To Opportunity) has been considered the “gold standard” in housing mobility research due to its experimental design, duration, and extensive data collection. Longitudinal research outcomes from MTO were a primary motivating factor for the establishment of Families Flourish in Central Ohio. Comparing the outcomes from Families Flourish to outcomes from MTO (and other similar mobility programs) remains difficult due to the heterogeneity of program design. For example, MTO utilized an experimental design (with a control group) in its design and approach; few other mobility programs throughout the United States have evaluated mobility through this methodological lens. Other mobility programs have been designed in response to fair housing litigation, with research evaluation occurring as an afterthought of what was primarily a legal remedy.

Despite these differences, some common themes do emerge. Early MTO qualitative research did identify mental health improvements for MTO families (similar to findings in Families Flourish). Similar to Families Flourish, improved outcomes for children and youth were also documented in the MTO research. The sharpest distinction between MTO outcomes and Families Flourish was related to economic outcomes for adults. MTO found no significant economic impact on adults in mobility programs. Families Flourish has demonstrated substantial economic gains in both the pilot program (run from 2018 to 2022) and the current program. We feel two distinct differences can be important in interpreting the economic gains in Families Flourish. First, MTO was utilizing federally subsidized housing vouchers. Vouchers have unique administrative requirements that Families Flourish avoids due to subsidies being privately funded. For example, the “benefit cliff” (where families risk losing housing assistance if their incomes rise too much) raises limitations for MTO; this issue is avoided in Families Flourish. Second, the life coaching program in Families Flourish is unique and did not exist in MTO or in other housing mobility programs. While more recent mobility programs have utilized “mobility counselors” to help movers navigate their new neighborhoods, the intensive life coaching model of Families Flourish is unique.

### 5.5. Limitations

There are several limitations to this study. First, Families Flourish is a program without a control or comparison group due to limited program capacity. As a result, we were unable to control for potential confounding factors, and the observed temporal patterns should be interpreted cautiously. Future research could strengthen the evidence base by incorporating an appropriate control or comparison group. Additionally, this program was implemented in the Columbus region and is currently expanding to other regions in Ohio. The region is characterized by economic segregation, conditions that are common in many metropolitan areas in the U.S. Accordingly, the findings may be most applicable to urban regions with similar housing conditions. Finally, Families Flourish offers housing assistance that is limited in both duration and amount, rather than long-term or deeply subsidized housing support. Although participating families were low-income and technically qualified for federal housing assistance, they were not as economically disadvantaged as the general population receiving rental housing assistance, such as those using a Housing Choice Voucher or living in public housing in the U.S.

## 6. Conclusions

Families Flourish participants reported a wide range of positive health outcomes, driven by safer housing, reduced stress, supportive coaching, improved financial stability, and access to better school environments. Together, these factors enabled families to promote healthier lifestyles, better manage chronic conditions, and enhance emotional well-being across generations. While individual experiences varied, the majority of participants described meaningful improvements in both mental and physical health, underscoring the program’s role in fostering holistic family well-being.

Children benefited from the Families Flourish program due to a simultaneous change within their housing unit, within the household and external neighborhood environment. The positive changes observed are consistent with Acevedo-Garcia et al.’s [14] theoretical conceptualization of housing mobility benefits for children. As demonstrated in earlier mobility research, improved neighborhood safety was a critical environmental shift that impacted outdoor activities, youth mental health, and parent mental health. Families Flourish parents demonstrated the same phenomena of living “life on watch” (as described by Kling et al.) in their former neighborhoods, constantly concerned about their children’s safety [17].

In alignment with the findings of Fauth et al. [19], Families Flourish families reported a shift in parenting style after relocating from their previous high-stress environments. Parenting was influenced by stress associated with housing insecurity, economic insecurity, and environmental risks. Positive shifts in parenting techniques were aided by life coaching and activities to support wellness among parents. The majority of children socially integrated into their new school environments without difficulty.

Housing mobility programs address two powerful environmental social determinants of health: housing unit quality and neighborhood quality. The outcomes that demonstrate health improvements for children in the Families Flourish program align with previous research documenting the positive health benefits of housing mobility. Through the longitudinally collected qualitative data, we were able to document the process of health improvement for families in the program.

Immediate improvements to indoor air quality and neighborhood safety improved respiratory health outcomes and facilitated more outdoor activity and play. Reduced family and child stress improved mental health outcomes for children and improved parenting style and parental engagement. Participation in a well-resourced school system provided more direct support from educators and supportive services for children. These underlying conditions would improve the socio-emotional health of children and school engagement. These foundational improvements in the environment directly resulted in positive educational outcomes for children.

In planning for family health, fair housing mobility programs are one specific tool that can improve health outcomes. The experience of Families Flourish indicates that improvement to children’s health and educational outcomes occurs sequentially over time. Our research suggests that a three-year subsidy may be sufficient to support housing stability and economic improvement for families in this study, particularly if they are not contending with ‘benefit cliff’ barriers. These findings suggest that longer-term housing subsidies are necessary to achieve long-term benefits. In contrast to transitional housing assistance, which may only last a few months, or vouchers, which are long-term, a three-year program with no limitations on increasing income could move families into a better economic position to navigate the private housing market without subsidy. The integration of extensive coaching and peer (family to family) engagement activities also provides critical social support for families who have relocated.

Fair housing mobility policies should complement other strategies that improve the overall supply of affordable healthy housing and planning efforts to improve environmental conditions and resources in disadvantaged neighborhoods. Fair housing mobility programs should also be aligned with efforts to reduce exclusionary housing barriers (particularly regulatory barriers such as exclusionary zoning) in safe, healthy, and highly resourced neighborhoods. More importantly, planning for family health requires prioritizing the agency and preferences of families marginalized by the housing market. The design of the Families Flourish program was informed by practice-based research outcomes but also by intensive engagement with marginalized families. Engagement activities sought to understand families’ preferences in regard to housing location, housing characteristics, and identification of neighborhood services (such as school system characteristics and transportation needs) that are a high priority for families.

## Figures and Tables

**Table 1:** Baseline participant characteristics (descriptive).

	Groups 1 and 2 (n = 27)	Groups 3 and 4 (n = 28)
Age, years (Median)	29	28
Household size (Median)	2	2
Estimated gross income (Median)	$34,900	$35,180
Credit score (Median)	539.5	588
Some post-secondary education (%)	61.5%	80.8%

Note. Values are reported descriptively as medians, or percentages, as appropriate, using available (non-missing) data; no statistical tests were conducted.

## Data Availability

The original contributions presented in this study are included in the article and Supplementary Material. Further inquiries can be directed to the corresponding author.

## Supplementary Materials

Supporting and background information for this study can be downloaded at: https://www.familiesflourish.org/reports (accessed on 11 May 2026). The following supporting information can be downloaded at: https://www.mdpi.com/article/10.3390/ijerph23060724/s1. Table S1: Within-participant changes in parent health outcomes between Year 1 and Year 2 (n = 24); Table S2: Within-participant changes in child health outcomes between Year 1 and Year 2 (n = 30).
