# Supplementary material for: Families Flourish: Triangulating Housing, Neighborhood, and Life Coaching for Health
Source: Int J Environ Res Public Health. 2026 May 29;23(6):724. doi: 10.3390/ijerph23060724 (PMC13299553; doi:10.3390/ijerph23060724)
Supplement: Supplementary file 1 [file ijerph-23-00724-s001.zip › ijerph-4185907-supplementary.pdf]

Supplementary Materials. Supplementary Within-Participant Analysis

This supplementary material presents descriptive within-participant comparisons for participants with data at both Year 1 and Year 2.

As supplementary context for the primary cohort-based findings, the supplementary within-participant analysis indicates that most participants reported stable or improved physical health, mental health, and stress level between Year 1 and Year 2. Among participants who reported changes over time, improvements were more frequently reported than worsening. Specifically, no participants reported worsening in mental health or stress at Year 2, while changes in physical health were more evenly distributed, with only one additional participant reporting worsening compared to improvement.

Table S1. Within-Participant Changes in Parent Health Outcomes Between Year 1 and Year 2 (n=24).

	**Physical health (n; %)***	**Mental health (n; %)**	**Stress level (n; %)**
Stable positive	11 (47.8%)	16 (66.7%)	14 (58.3%)
Improved	3 (13.0%)	7 (29.2%)	7 (29.2%)
No change	4 (17.4%)	0 (0%)	2 (8.3%)
Worsened	4 (17.4%)	0 (0%)	0 (0%)
Stable negative	1 (4.3%)	1 (4.2%)	1 (4.2%)

*One item-level response was missing; calculations are based on available (non-missing) data.

Similarly, for children's outcomes, most participants reported stable or improved physical health, mental health, emotional health, and behavior between Year 1 and Year 2. Among participants who reported changes over time, improvements were more commonly reported than worsening across all child health outcomes. These supplementary findings are descriptive and should be interpreted in light of the sample size and absence of a control group.

Table S2. Within-Participant Changes in Child Health Outcomes Between Year 1 and Year 2 (n=30).

	**Physical health (n; %)**	**Mental health (n; %)**	**Self-image (n; %)**	**Optimism (n; %)**	**Behavior (n; %)**
Stable positive	11 (36.7%)	10 (33.3%)	12 (40.0%)	11 (36.7%)	15 (50.0%)
Improved	9 (30.0%)	8 (26.7%)	8 (26.7%)	8 (26.7%)	8 (26.7%)
No change	6 (20.0%)	7 (23.3%)	8 (26.7%)	7 (23.3%)	5 (16.7%)
Worsened	3 (10.0%)	5 (16.7%)	2 (6.7%)	4 (13.3%)	2 (6.7%)
Stable negative	1 (3.3%)	0 (0%)	0 (0%)	0 (0%)	0 (0%)
